# The Evolution of Heterogeneities Altered by Mutational Robustness, Gene Expression Noise and Bottlenecks in Gene Regulatory Networks

**DOI:** 10.1371/journal.pone.0116167

**Published:** 2014-12-26

**Authors:** Zhihua Zhang

**Affiliations:** Chinese Academy of Sciences Key Laboratory of Genome Sciences and Information, Beijing Institute of Genomics, Chinese Academy of Sciences, Beijing, China; Leibniz-Institute for Farm Animal Biology (FBN), Germany

## Abstract

Intra-population heterogeneity is commonly observed in natural and cellular populations and has profound influence on their evolution. For example, intra-tumor heterogeneity is prevalent in most tumor types and can result in the failure of tumor therapy. However, the evolutionary process of heterogeneity remains poorly characterized at both genotypic and phenotypic level. Here we study the evolution of intra-population heterogeneities of gene regulatory networks (GRN), in particular mutational robustness and gene expression noise as contributors to the development of heterogeneities. By *in silico* simulations, it was found that the impact of these factors on GRN can, under certain conditions, promote phenotypic heterogeneity. We also studied the effect of population bottlenecks on the evolution of GRN. When the GRN population passes through such bottlenecks, neither mutational robustness nor population fitness was observed to be substantially altered. Interestingly, however, we did detect a significant increase in the number of potential “generator” genes which can substantially induce population fitness, when stimulated by mutational hits.

## Introduction

Living organisms are always exposed to external and internal perturbations. In most situations, these perturbations do not have observable effects, essentially because living organisms are robust systems adapted to their environments. However, in some cases, these perturbations, such as genetic mutation or nongenetic gene expression noise, create novel phenotypes. The frequency of these novel phenotypes may be changed in the population during the process of evolution, thus changes genotypic and phenotypic heterogeneities within the population. Change in heterogeneity is universally observed in both natural and cellular populations. For example, among tumor cells, the most advanced malignant tumors remain incurable as a result of resistance to therapy, relapse or metastasis, and the large increase in intra-tumor heterogeneity is cited as the cause [1], [2]. However, the evolutionary process of such population of heterogeneities remains poorly characterized. In nature, most genetic variations may be neutral in a population. Consequently, genotypic and phenotypic heterogeneity of a population is, in general, not linearly correlated [1]. Therefore, to quantitatively study heterogeneities, it is necessary to distinguish genotypic from phenotypic heterogeneities and then connect the two with a measureable model. Several systems have been used as computational models for this purpose, including RNA secondary structure, protein structure and gene regulatory network (GRN) [3]. In this paper, we studied the dynamics of genotypic and phenotypic heterogeneities in a population of GRNs. Currently, we lack qualified data and tools for quantitative models of a network, even for small networks. Alternatively, the qualitative binary model has been proven to be a powerful tool for the study of complex systems, and many basic evolutionary principles have been revealed through such systems [4], [5].

In addition to genetic variations, epigenetic variations [6] and stochastic fluctuation in gene expression, or the so-called gene expression noise [7], can also contribute to phenotypic variation in a cell population. As such variability is inheritable between cell generations [8], gene expression noise could be a driving force for adaptation while under stress [9], and it has also been suggested to play a role in the development of heterogeneity during the early stages of tumorigenesis [2], [10]. On the other hand, the effect of both gene expression noise and genetic perturbations may be buffered by the robustness of the system. The associations between robustness, gene expression noise and phenotypic variability have been addressed in a binary GRN system [11], which revealed that mutational robustness is evolvable in the GRN system [12]. However, in this GRN system, it remains unclear if interactions occur between mutational robustness and internal/external perturbations during the evolutionary development of heterogeneities. In addition, the environmental changes also has effect on the response in evolutionary process of phenotypic variability. This effect has also be investigated by the binary GRN system and has been shown with conjoint variability between phenotypic robustness and phenotypic variability after environmental changes [11]. However, in nature, a kind of force majeure can occur at any time in the environment, such as unexpected disasters, resulting in a population bottleneck in the evolution of a living population. In the context of the present work, we still do not know how population bottlenecks in the evolution of GRNs can trigger changes in heterogeneities.

To address these questions, we adopted a simple binary GRN model as a tool to investigate dynamic changes of heterogeneities. This network model was developed by A. Wagner [13], and it has been applied to many studies of evolutionary biology [13]–[15]. Briefly, a GRN is a set of genes and their protein products together with the regulatory relationship between them. A GRN can be represented by a conjunction matrix *M,* where each row and column represents a gene (together with it protein product), and each element represents the regulatory relationship between the genes in the corresponding row and column. The expression status of genes, i.e. silenced or expressed, in the network is represented by a binary vector in which -1 and 1 imply silenced and expressed genes, respectively. The dynamic of this system is determined by formula (1) (see the Method and Materials). As the topology of this network is presumably defined by the promoter sequences of the genes, the topology *per se* can be regarded as the genotype of the cell carrying this network, and the steady state of gene expression pattern in this network can be regarded as the corresponding phenotype [12], [13], [15]. With this genotype-to-phenotype mapping, a population of networks was subjected to *in silico* evolution for an ensemble of parameter settings. The phenotypic heterogeneity was indexed as the compositional diversity of gene expression patterns in a network population. We first demonstrated that this network model is a simple, but powerful, tool for studying the evolution of heterogeneity in a network population, and with the help of gene expression noise and high mutational robustness, it was found that a GRN can promote phenotypic heterogeneity under certain conditions. We then showed that population bottlenecks do not affect overall phenotypic heterogeneity. However, when we looked the heterogeneities at another level, *i.e.*, the number of possible “generator” genes, which may be defined as a gene that substantially induces population fitness while being hit by mutations, we found significant increases in the number of “generator” genes post-bottleneck.

## Results

To investigate the evolutionary process of genotypic (GH) and phenotypic (PH) heterogeneities, we adopted a simple binary GRN model to map genotype and phenotype [13]. The heterogeneities were measured as the Shannon entropy in a population (see details in Method and Materials).

### GH and PH of GRN are affected by mutation rate and population size

By the nature of Shannon entropy, the maximal entropy level of a network population strongly depends on the maximum number of network states it has. In a population with a finite number of individuals, when no pair of individuals carries identical genotypes/phenotypes, the population reaches the upper limit of heterogeneity. When the total number of all possible network states is larger than the population size, *i.e.*, the number of individuals in the population, this upper limit of entropy is the function of population size. For a given binary network with *N* genes, when the population size is smaller than 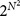, the upper limit of genotypic heterogeneity solely depends on population size. Thus, to make a fair comparison of heterogeneities between populations, we only compared the entropies of populations with identical population size. Since the phenotypic space is a *N*-dimensional space embedded in the 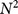-dimensional genotypic space, it is possible that no pair of individuals in the population will carry identical genotypes when the population size is too small, which will make the PH level less than the upper limit. Even if the population size is larger than 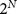, the PH will still be less than its upper limit. To avoid such situations, we set the gene number *N* be 20, and we chose population size as 50, 100, and 200 so that both PH and GH would reach the upper limit in the condition of neutral mutations (S1A Fig.), and when the mutations are not neutral, both GH and PH will be much less than their upper limits (S1B Fig.).

**Figure 1:**
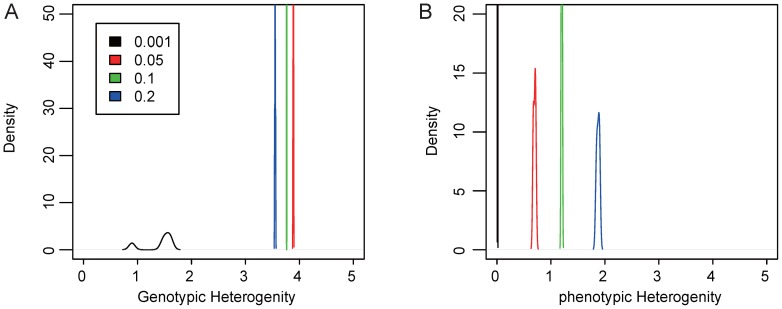
Heterogeneity levels when a population is subjected to stabilizing selection. This figure shows the density of converged heterogeneity levels from 50 replications of simulation for a population with 100 individuals. The colors represent the mutation rates. A) genotypic heterogeneity and B) phenotypic heterogeneity.

When a network population is subjected to stabilizing selection, we asked what the effect of mutation rate and population size would be toward the dynamic of genotypic and phenotypic heterogeneity (see Method and Materials). Simulations were performed with a group of parameter settings (S1 Table). Except for the case when mutation rate is extremely low (*e.g.*, 0.001), we found GH to slightly decrease when mutation rate increases, while PH substantially increases (Fig. 1). This phenomenon can be explained by the metagraph property of networks. The metagraph (also called metanetwork) was introduced by A. Wagner [12], in a network metagraph, each node represents a network, and each link between two nodes indicates that the two connecting nodes differ by only one regulatory interaction [12]. When mutation rate is extremely low, such as 0.001 in our simulation, chances are small (*i.e.*, by a factor of 0.2 networks) that a network will be hit by more than two mutations within a generation. In that case, most mutations will either be lethal or produce the same phenotype, since it has been demonstrated that all, or most, networks with the same phenotype form a connected metagraph in the genotype space [12]. For a few mutations that do generate new phenotypes, these new phenotypes are also subjected to random drift. This explains why GH and PH are both low in simulations where mutation rate is extremely low. However, when the mutation rate is not so low, for example 0.05 in a population with 200 individuals, a 50% chance exists that an individual could be hit twice in one generation. The double hits could easily be lethal to a network, and with higher mutation rate, more individuals would be at risk of multiple hits which would, in turn, reduce GH. On the other hand, the total number of individuals who experience such multiple hits would be considerably lower than the population size, thus decreasing the chance of heterogeneity. Since phenotype space is only a *N*-dimensional space embedded in a 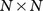-dimensional genotype space, the overall PH level is much lower than the GH level. Under these conditions, more mutations introduced by a higher mutation rate will result in access to more phenotypes in the phenotypic space and, hence, higher PH levels.

### Convergent evolution of mutational robustness of GRNs

**Figure 2:**
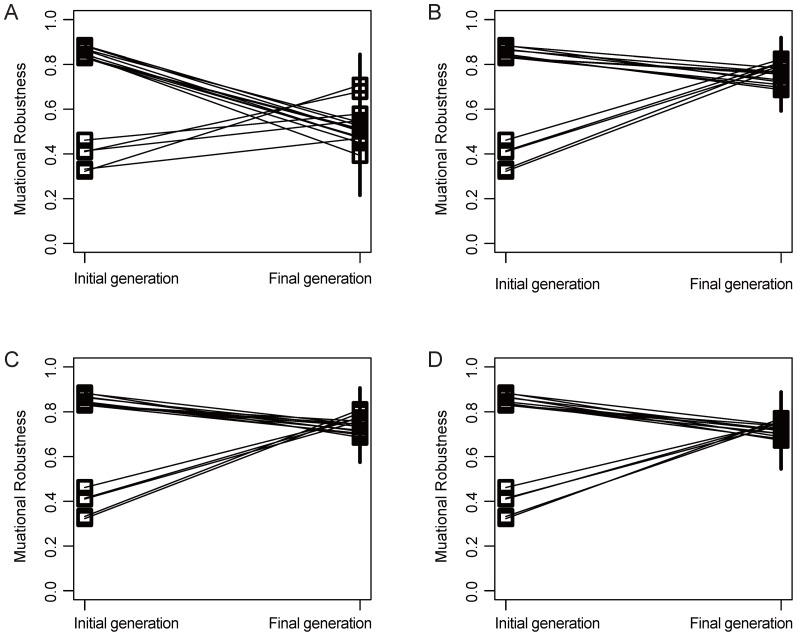
Convergent evolution of mutational robustness. The dots represent the actual mutational robustness levels of a group of GRNs having identical initial *d* = 0.1. The mutational robustness levels at the initial and final generation are linked by solid lines. The population size is 100, and mutation rates are A) 0.001, B) 0.05, C) 0.1 and D) 0.2.

In all the simulations we examined, the networks will ultimately converge to a genotype population whose mutational robustness is about 0.6∼0.8. Previously, it has been suggested that 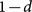, where 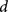 denotes the fraction of genes whose expression status between 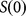 and 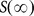, could be used as an agent for mutational robustness [5], [11]. However, we did not find this to be a fine-tuned index. For example, in the networks we examined with 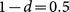, the average actual mutational robustness was 0.85, while for the networks with larger 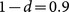, the average actual mutational robustness was only 0.81, which is significantly smaller than 0.85 (Student's *t*-test, *P*-value  =  3.1E-10). The parameter 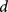, however, indicates the level of converged mutational robustness (Fig. 2 and S2 Fig.). In the simulation we performed, the final mutational robustness of populations after stabilizing selection could be clearly classified into groups according to the initial 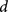 value. A common characteristic shared by most members of each group is the *d* value in their initial genotype; therefore, 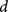 can be used as a proxy for converged mutational robustness after stabilizing selection. Under the condition of natural selection, it has been previously shown that a large population with sufficiently high mutation rates (*i.e.*,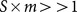, where 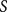 denotes population size and *m* denotes mutation rate) becomes concentrated at genotypes of higher mutational robustness than average [16]. In particular, the average mutational robustness of this large population will converge to an eigenvalue associated with the adjacency matrix of the metagraph [17]. This result was based on biological macromolecules, and it can also be applied to the network model. Given this evolving nature of the GRN, the term mutational robustness will hereinafter only refer to the final converged level of mutational robustness, unless otherwise noted.

### The interaction between gene expression noise and mutational robustness as a factor of PH induction

In addition to population size and mutation rate, we asked if the PH could also be affected by the genetic property of the genotype *per se*. Mutational robustness is a widely discussed GRN property. In a large scattered viable network, easy access to innovative phenotypes during evolution has been suggested by the availability of highly robust mutations [12], [15]. However, we have not fully elucidated the likelihood that this innovation could be conserved and spread in a population [11].

Another factor complicates this picture, the gene expression noise. Gene expression noise is defined as stochastic fluctuation that results from intrinsic and extrinsic perturbations which could occur at both transcription and translational levels. The immediate observation of gene expression noise is a diverse expression levels in isogenic population under identical experimental conditions. As extrinsic perturbations are not consistent from condition to condition, we only considered intrinsic gene expression noise in this study and modeled it as an additive perturbation (See Methods and Materials). Intrinsic gene expression noise is an inheritable genetic characteristic of gene expression [18], [19], and can induce adaptation under certain conditions [9]; however, we still do not know how this adaptive driving force can influence PH during the process of evolution. Moreover, as shown by Cilliberti and colleagues [15], mutational robustness is weakly correlated with robustness to noise. Therefore, key questions emerging from these observations are whether any interaction exists between mutational robustness and intrinsic noise, and, if so, how much will such interaction contribute to the development of PH.

To address these questions, we employed a simple linear regression model in a series of conditions of given mutation and population size (Table 1). In all conditions we examined, the final PH that a population reached could be properly modeled by mutational robustness and gene expression noise, in which the coefficients for robustness are consistently negative, while the coefficients for noise are consistently positive. Since a population with highly phenotypic robust genotype will be less heterogeneous after stabilizing selection, this result may be considered intuitive. It is also a result that contradicts the notion that networks with higher mutational robustness have a greater chance of reaching new phenotypes, as long as the metanetwork of this genotype locus has a sufficiently large diameter [12], [15]. Nonetheless, this result does agree with that of Espinosa-Soto, et al. (2011) who showed that PH, as represented by phenotypic variability, is responsive to nongenetic perturbations, such as those that occur in the environment, but unresponsive to mutations [11].

**Table 1:** Coefficients in the linear regression models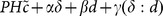.

	Population size								
*_m_*	50			100			200		
	*α*	*β*	*γ*	*α*	*β*	*γ*	*α*	*β*	*γ*
0.001	−0.023**	0.012	0.020**	−0.014**	0.085	0.023*	−0.006	0.065	0.043**
0.05	0.020**	0.631**	−0.000	0.016**	0.748**	0.006	0.030**	0.850**	−0.015*
0.1	−0.017**	0.566**	0.010	−0.020**	0.629**	0.012	0.015**	0.941**	−0.046**
0.2	−0.043**	0.408**	0.023**	−0.042**	0.601**	0.003	−0.012**	0.821**	−0.028**

The interaction between gene expression noise (*δ*) and mutational robustness (*d*) is considered (*δ*:*d*). The coefficients significantly not zero (F-test) are marked by * (P-value <0.01) and ** (P-value <0.0001).

**Figure 3:**
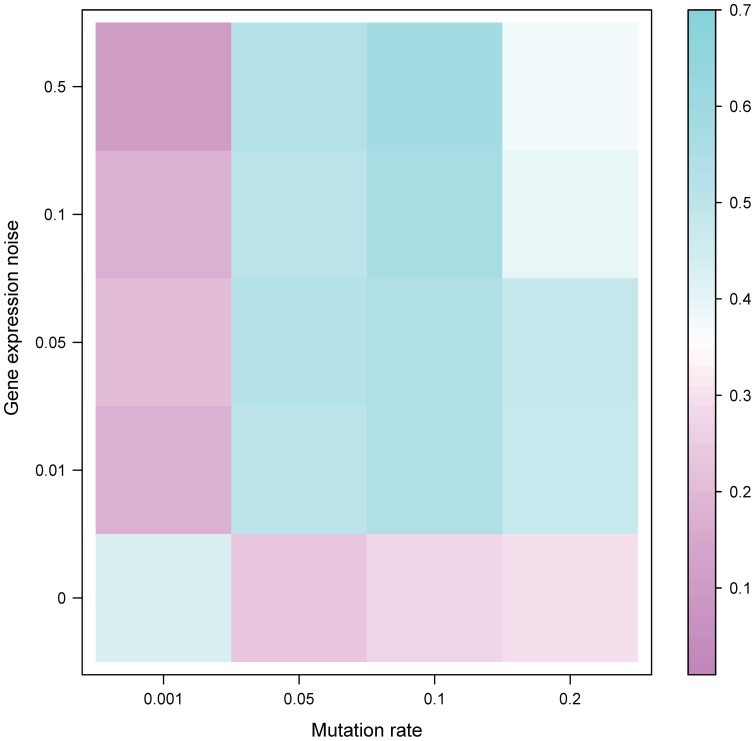
The interaction among mutation, mutational robustness and gene expression noise. Each square represents a set of simulations with identical gene expression noise level and mutation rate. The color in each square index *_R_* was defined as the proportion of simulation pairs indicating that a population with higher mutational robustness would be more phenotypically heterogeneous than a population with lower mutational robustness after stabilizing selection. The population size is 100.

To further investigate the landscape of interactions between mutational robustness and PH, we conducted the following survey. For any given mutation rate and noise level, we evaluated the likelihood that populations with higher mutational robustness would be more phenotypically heterogeneous than populations with lower mutational robustness under stabilizing selection. To quantify this postulate, we denoted the proportion of simulation pairs satisfying the above conditions from all pairs of simulations as *R*, as shown in Fig. 3 and S3 Fig., and *R* was shown to be small in the following two scenarios, irrespective of population size: 1) when mutation rate is extremely low (*e.g.,* 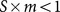) and the noise level is not zero and 2) when noise level is zero and mutation rate is not too low (*e.g.,* 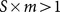). Interestingly, when mutation rate is extremely low and no expression noise is involved, the evolutionary process is similar to a random walk in the metanetwork whose topology determines the outcome of PH. Therefore, we observed an approximate 0.5 in *R*, irrespective of population size. However, when both mutation rate and gene expression noise levels are considerably larger than zero, such random walking in the metanetwork is no longer a proper analogue because the phenotype of a network is less predictable, and the networks are less likely be viable [12], [15]. Under those scenarios, we observed significantly larger *R* than the single factor scenarios we discussed above, and *R* is also approximately 0.5, irrespective of population size (Fig. 3 and S3 Fig.). These results, in addition to the linear model (Table 1), imply that PH, at least in the range of parameters we simulated, cannot be induced by either genetic mutation or gene expression noise alone; instead, it is the the interaction between the two and phenotypic robustness that can significantly induce PH.

### Population bottlenecks do not affect mutational robustness and PH in network populations

In nature, unforeseen, dramatic events can cause equally dramatic alterations in the evolutionary paths of populations. To study such effects on populations, we modeled the events as evolutionary population bottlenecks. A population bottleneck is an event that, at least for one generation, the size of a population has be drastically reduced. One type of population bottleneck (type I) randomly removes individuals, irrespective of their genotypes. This type of population bottleneck is used to mimic such events as earthquakes which may kill nearly all animal and plant life in a given region. Another type of population bottleneck (type II) targets one or more major clones. This type of population bottleneck is analogous to the effect of chemo- or radiotherapy on tumor cells. To simulate type I, we randomly removed a given percentage of cells from the population, while to simulate type II, we removed genotypic clones in the reverse order of size until a given percentage cells of the population was left.

We then asked if the average mutational robustness and PH of a population could be altered after passing through such population bottlenecks. In the parameter space we sampled, we allowed the populations to evolve through both type I and II population bottlenecks. After the populations passed through these bottlenecks, we let them continue to evolve with the same number of pre-bottleneck generations. Average mutational robustness and PH level were compared at two time points: right before the population bottleneck and after the last generation simulated. For PH, except for cases of extremely low mutation rate, we did not observe any significant difference between the two time points (S2 Table). Similarly, although we did not observe any change in mutational robustness post-bottleneck (S4 and S5 Figs.), an exceptional case arose when a population passed through a type II population bottleneck without the involvement of gene expression noise (S5 Fig.). However, even in this case, although the difference is statistically significant, only weak changes in absolute numbers were observed, and such a small change in average mutational robustness post-bottleneck failed to translate into the final PH.

### Population bottlenecks induce the generation of novel potential “generator” genes

**Figure 4:**
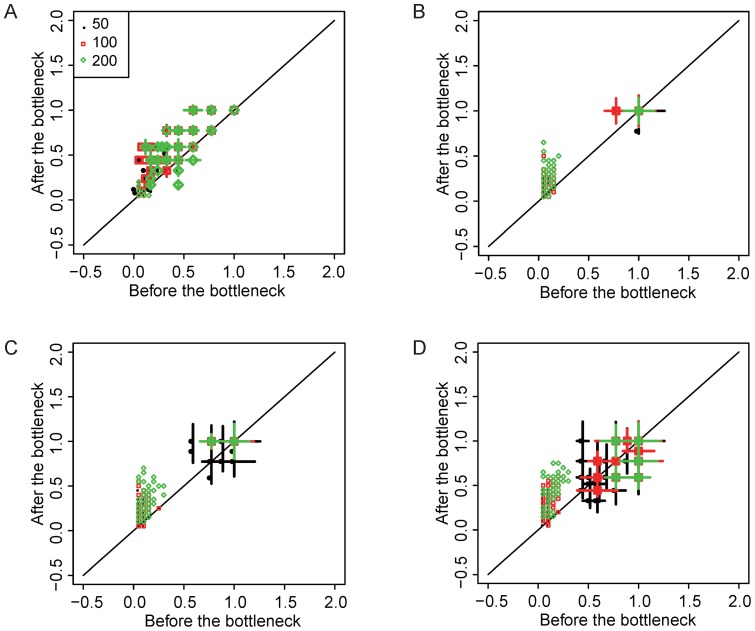
Fitness gain and number of potential oncogene changes when subjected to type I population bottleneck. Each boxplots represents median and standard deviation of maximal fitness gain before and after the bottleneck for 50 simulation repeats. The dots represent the percentage of potential oncogenes before and after passing through the bottleneck (see main text for definition). Gene expression noise level is 0, population size is indicated in color, and mutation rates are A) 0.001, B) 0.05, C) 0.1and D) 0.2.

Entropy measures the overall divergence of a given population. However, the contributions of mutations found in a population to the evolutionary process are not identical. Besides viable mutations, nonviable mutations also cause differences in fitness gain or loss. In the context of cancer, a huge number of mutations have been reported, *e.g.,* in The Cancer Genome Atlas (https://tcga-data.nci.nih.gov/); yet, most such mutations are believed to be passengers, while only a few have been identified as drivers. Therefore, it is more interesting to investigate the divergence of those key genes which can substantially influence fitness of a cell after having been hit by mutations. In this study, we called all those key genes which can reach maximal fitness gain when hit by mutations as “generator” genes. We sought to compare the number of unique “generator” genes before and after population exposure to the two types of population bottlenecks. By definition, each network contains at least one “generator” gene. In a homologous population, “generator” genes will also be homologous, while in a population with more divergent potential, the “generator” genes will be heterogeneous. We compared the number of unique “generator” genes in a population before and after passing through the population bottlenecks. For both types of population bottlenecks, the number of unique “generator” genes significantly increased, irrespective of mutation rate, population size or gene expression noise level (Fig. 4, S6, S7 and S8 Figs.).

To account for this observation, the network from minor clones might have passed through the bottleneck stochastically, and those networks might have been located at a distal region in the genotype far from the network of the dominant clone. Post-bottleneck, however, new start points may arise from this distal region, resulting in population fitness with a correspondingly better chance to reach a more adaptive genotype than that of the dominant clone. If this hypothesis is true, we would expect an increase in the number of unique “generator” genes and the average possible maximal fitness gain post-bottleneck. To test this, maximal potential fitness gains from all possible mutations in networks were calculated. For both types of bottlenecks, a significant maximal fitness gain was observed in most scenarios (Fig. 4, S6, S7 and S8 Figs.). However, this fitness gain will have no influence over the evolutionary pattern of the model with fixed population size, as only relative fitness affects the selection process. This also explains why we did not detect substantial changes in either mutational robustness or PH post-bottleneck. In an analogous *in vivo* context, tumor growth would not be limited by a pre-assigned population number, but rather by the space or resources it can access. In other words, without any additional genetic or microenvironmental assumptions, this result demonstrated that a population could proliferate more aggressively after having passed through a population bottleneck than before such passage. Moreover, as indicated by the larger number of unique “generator” genes after passing through population bottlenecks, the drivers of proliferation post-bottleneck could be more heterogeneous.

## Discussion

We studied the evolutionary process of a GRN model of genotype and phenotype in a cell population and observed interactions among mutation, gene expression noise, mutational robustness and phenotypic heterogeneities. It was revealed that gene expression noise and mutational robustness, apart from population size and mutation rate, are two key factors that drive the evolution of PH. We detected strong interactions among mutational robustness, gene expression noise and mutation rate. By simulation, we also studied the dynamics of bottleneck effect on phenotypic heterogeneity and detected a strong increase in the number of potential “generator” genes, when stimulated by mutational hits.

The method presented in this paper highlighted the usefulness of applying the GRN approach (see A Wagner 1994) to understand the general evolutionary process of heterogeneities, a subject which has been largely overlooked [20]–[22]. At the same time, however, our model has been simplified from the real evolutionary process of heterogeneities in several respects. First, the total population numbers we simulated were several orders of magnitude smaller than actual effective population size in real organisms or cellular populations [23], as a result of the limitations imposed by computational capacity. Moreover, this work considered a population with fixed population size. It has been demonstrated that the growth of populations contributes to the increase of heterogeneities [24]. Because we are interested in the factors that contribute to PH, other than population growth, we controlled the effect of growth by setting a fixed population size. Second, we assumed relatively high mutation rates compared to actual mutation rates observed in nature [25], [26]. We did this because population sizes in our simulations were several orders of magnitude smaller than those seen in nature; thus, relatively high mutation rates could result in accumulating sufficient variations in a population to, in turn, affect PH. For example, an analogy might be found in a cellular population undergoing tumor metastasis in which a very high mutation rate may exist at certain stages of evolution [27]. Thus, our high mutation rate scenario could partially mimic this situation. Furthermore, we assumed an evenly distributed mutation rate for all genes in the networks, however, it was suggested that mutation rate could vary across genome [28]. Third, we assumed identical gene expression noise level in the entire GRN. In real living organisms, gene expression noise level has been associated with the importance of the genes [29]. Because not all genes are equally important, the gene expression noise level varies between genes [30]. In our simulations, as we did not specify any particular sequence in the imaginary promoters, and because of the limited number of genes in the networks, the importance of a gene cannot be realistically linked to any network property, such as connectivity, in turn, it is not plausible to have unequal expression noise level assignment. However, this assumption may not be an oversimplification, as randomly altered a few gene expression noise levels in the simulations did not substantially change the conclusions (data not shown). More realistic models to take consider in these assumptions await further scrutiny.

In the present study, two types of population bottlenecks were discussed, each one having its analogue in nature. In type I population bottleneck, the determination of survival individuals is purely by chance, a consequence which would have profound effects on population structure or even speciation [31]. While in type II population bottleneck, we targeted individuals with particular geno- or phenotypes for removal. There are many environmental changes which may be resulted in reducing population size, and physiological response to those changes may not identical from one to another [32], since we are interested in common principle of the evolutionary process of heterogeneity and since the network model is not intended to mimic any particular living organisms, we have omitted the details of all possible environmental catastrophes.

In this study, we introduced the concept of “generator” genes and defined them as genes able to reach maximal fitness gain when hit by mutations. “Generator” genes also have analogues in nature. For example, in the context of carcinogenesis, oncogenes have long been identified as the underlying drivers for clonal expansion when hit by mutations [33]. The mechanisms by which individual mutant oncogenes promote carcinogenesis are remarkable variable [33]. On the other hand, although an enormous number of passenger mutations have been identified in nearly all tumor types (https://tcga-data.nci.nih.gov/), with the exception of a few driver mutations, most of these passenger mutations will have no visible phenotypic consequence. Therefore, the number of mutant oncogenes, representing the number of altered signaling pathways, provides yet another key layer of heterogeneity in a tumor cell population.

The identity of “generator” genes is dependent on the fitness landscape which was artificially defined in this work. Although the landscape of fitness based on cumulative mutations in the context of bacteria and viruses has been experimentally studied [34], [35], few studies have reported on the fitness landscape of phenotypes in other systems, *e.g.,* mammalian cells. We adapted a fitness landscape from Espinosa-Soto *et al.* [11], which assumed an optimal expression pattern after altering the environment. However, our results do not completely depend on the specific form of landscape because an alternative fitness landscape (defined as 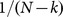 data not shown) generated similar results.

In conclusion, the evolution of heterogeneity in GRN is a complicated process which depends on a wide range of conditions, including genetic factors, *e.g.,* mutational robustness, intrinsic gene expression noise, as well as non-genetic factors, *e.g.,* population size and the bottlenecks it experiences. Although we based our observations of a simple GRN model, we noticed similarities to many biological systems. However, it remains to be elucidated whether such observations will hold in other systems [3].

## Method and Materials

### Model description

We adopted a well-studied GRN model introduced by A. Wagner and designed to link a genotype to a phenotype [13]. Briefly, a network with 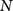
nodes represents the genotype of an individual with 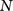 genes, in which the directional connections between the nodes represent regulatory relationships between the genes. The dynamic of this network was then defined by the following equation
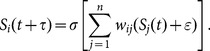
(1)


where 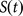  =  (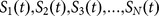) is the vector which contains all the gene expression states in the network at time point 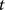, and 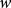 is the adjacency matrix of the network. The elements in this matrix are 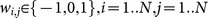. We introduced gene expression noise as an additive Gaussian white noise 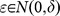, where 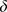 was used to represent the noise level, and 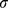 is the sign function, *i.e.,* 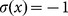 when 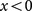 and 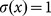 when 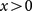. Therefore, the steady state 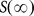
defines the phenotype of a given network w, given an initial gene expression vector 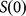. The sample process for network structure and the method of choosing initial and steady states are identical to [12].

In present work, we considered networks with 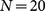, and for each 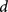 value (defined as the proportion of nodes in a network showing different expression level between initial and steady states), we sampled 10 networks. We only considered mutation hits in the promoter region of a gene, followed by one of the following consequences: 1) altering its regulatory behavior with equal probability, 2) gaining a new regulatory connection from a random gene in the network, 3) losing a random regulatory connection, or 4) changing the attitude of a random regulatory connection, *i.e.,* switching from an active regulation to a repressive one. The definition of mutational robustness for a network is from [12], *i.e.,* the fraction of one-mutant neighbors that is also viable.

#### Simulation of the evolution process

As described in [12], 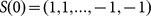 is composed of 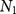 copies of *1* and 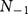 copies *of -1,* and 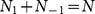. 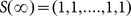 is composed of 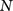 copies of 1. At any given environment, an optimal expression pattern arises, 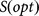, which yields the highest growth rate for an individual with the phenotype. However, for individuals whose phenotype is different from 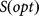, the fitness is defined as 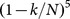, where *k* denotes the number of genes expressing differently from 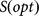, and the survival probability of this individual was set to the relative fitness in the whole population [11]. In all simulations, 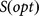 was set as 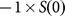 for stabilizing selection.

For the evolution process, we assumed no overlaps between generations, and we started with an isogenic population with 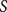 individuals, *i.e.,* 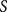 copies of initial networks. Random genes in random individuals were drawn from the previous generation for 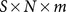 times, where 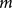 denotes mutation rate, and those genes were subject to mutations. After mutation, new relative fitness was recalculated for each genotype, including the new genotypes introduced by the mutations. The number of each genotype in the next generation is then proportional to the new relative fitness. The entire evolutionary process was implemented by Fortran90. For each combination of parameters (S1 Table), the simulation was processed for 100,000 generations and repeated for 50 times.

#### Measures of heterogeneity

Heterogeneity is measured by 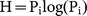, where 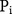 denotes the probability of the *i*-th genotype or phenotype in a population. It was approximated by the frequency of the *i*-th genotype or phenotype observed in the population. The genotype in this study refers to the network topology, in another word, the 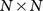
dimensional conjunction matrix of a given GRN. The phenotype refers the *N*-dimensional vector of expression states of all genes in a given GRN.

#### Two types of population bottlenecks

Type I population bottleneck involved an operation in which we randomly removed a given percentage of networks from the population, irrespective of the origin of clones. For type II population bottleneck, we removed networks from genotype clones in the reverse order until a given percentage cells of the population was removed. After a population passed through a bottleneck, we did not simulate the exponential growth process; instead, we calculated the relative fitness of remaining cells and made a new generation with 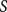 individuals according to the relative fitness.

## Supporting Information

S1 Fig **Evolutionary process of genotypic (GH) and phenotypic (PH) heterogeneities in an** ***in silico*** **simulation.** The population size in the simulation is 100, mutation rate is 0.1, mutational robustness is 0.1 and noise level is 0.1. A). When no selective constraint was involved, both GH and PH could reach to the upper limit of heterogeneities for the given population, but when the population was subjected to stabilizing selection B), the maximal heterogeneities for both genotype and phenotype were both substantially reduced, and PH was much less than GH.
(TIF)

S2 Fig **Convergent evolution of mutational robustness of GRNs.** The dot pairs linked by solid lines represent the actual mutational robustness at the beginning and end of simulations for a group of GRNs with identical population sizes. The population size is 50, and mutation rates are A) 0.001, B) 0.05, C) 0.1 and D) 0.2. Population size is 200, and mutation rates are E) 0.001, F) 0.05, G) 0.1 and H) 0.2.
(TIF)

S3 Fig
**The interaction among mutation, mutational robustness and gene expression noise.** Each square represents a set of simulations with identical gene expression noise level and mutation rate. The color in each square index 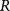 was defined as the proportion of simulation pairs indicating that a population with higher mutational robustness would be more phenotypically heterogeneous than a population with lower mutational robustness after stabilizing selection. The population size is A) 50 and B) 200.(TIF)

S4 Fig **The effect of type I population bottleneck on the evolution of mutational robustness of GRNs.** The dots linked by solid lines represent the actual mutational robustness at the beginning generation, before introduction of the bottleneck and after finishing simulations for a group of GRNs with identical population sizes. Population size is 50, and mutation rates are A) 0.001, B) 0.05, C) 0.1 and D) 0.2. Population size is 100, and mutation rates are E) 0.001, F) 0.05, G) 0.1 and H) 0.2. Population size is 200, and mutation rates are I) 0.001, J) 0.05, K) 0.1 and L) 0.2.
(TIF)

S5 Fig **The effect of type II population bottleneck on the evolution of mutational robustness of GRNs.** The dots linked by solid lines represent the actual mutational robustness at the beginning generation, before introduction of the bottleneck and after finishing simulations for a group of GRNs with identical population sizes. The population size is 50, and mutation rates are A) 0.001, B) 0.05, C) 0.1 and D) 0.2. Population size is 100, and mutation rates are E) 0.001, F) 0.05, G) 0.1 and H) 0.2. Population size is 200, and mutation rates are I) 0.001, J) 0.05, K) 0.1 and L) 0.2.
(TIF)

S6 Fig **Fitness gain and number of potential oncogene changes before and after passing through type II population bottleneck.** Each boxplots represents median and standard deviation of maximal fitness gain before and after the bottleneck for 50 simulation repeats. The dots represent the percentage of potential oncogenes before and after the bottleneck (see main text for definition). Gene expression noise level is 0, population size is indicated in color, and mutation rates are A) 0.001, B) 0.05, C) 0.1 and D) 0.2.
(TIF)

S7 Fig **Fitness gain and number of potential oncogene changes before and after passing through population bottlenecks.** Each boxplot represents median and standard deviation of maximal fitness gain before and after the bottleneck for 50 simulation repeats. The dots represent the percentage of potential oncogenes before and after the bottleneck (see main text for definition). Gene expression noise level is 0.01, and population size is indicated in color. For type I bottlenecks, the mutation rates are A) 0.001, B) 0.05, C) 0.1 and D) 0.2. For type II bottlenecks, the mutation rates are E) 0.001, F) 0.05, G) 0.1 and H) 0.2.
(TIF)

S8 Fig **Fitness gain and number of potential oncogene changes before and after passing through population bottlenecks.** Each boxplot represents median and standard deviation of maximal fitness gain before and after the bottleneck for 50 simulation repeats. The dots represent the percentage of potential oncogenes before and after the bottleneck (see main text for definition). Gene expression noise level is 0.5, and population size is indicated in color. For type I bottlenecks, the mutation rates are A) 0.001, B) 0.05, C) 0.1 and D) 0.2. For type II bottlenecks, the mutation rates are E) 0.001, F) 0.05, G) 0.1 and H) 0.2.
(TIF)

S1 Table **The parameters used in the simulations.** All the networks were generated with 20 genes, and for each combination of parameters, the simulation was processed for 100,000 generations and repeated 50 times.
(DOCX)

S2 Table **PH Difference between pre- and post-bottlenecks in GRN networks.** P-values were given by *t*-test.
(DOCX)
