# Supplementary figures and images for: The Evolution of Heterogeneities Altered by Mutational Robustness, Gene Expression Noise and Bottlenecks in Gene Regulatory Networks
Source: PLoS One. 2014 Dec 26;9(12):e116167. doi: 10.1371/journal.pone.0116167 (PMC4277480; doi:10.1371/journal.pone.0116167)

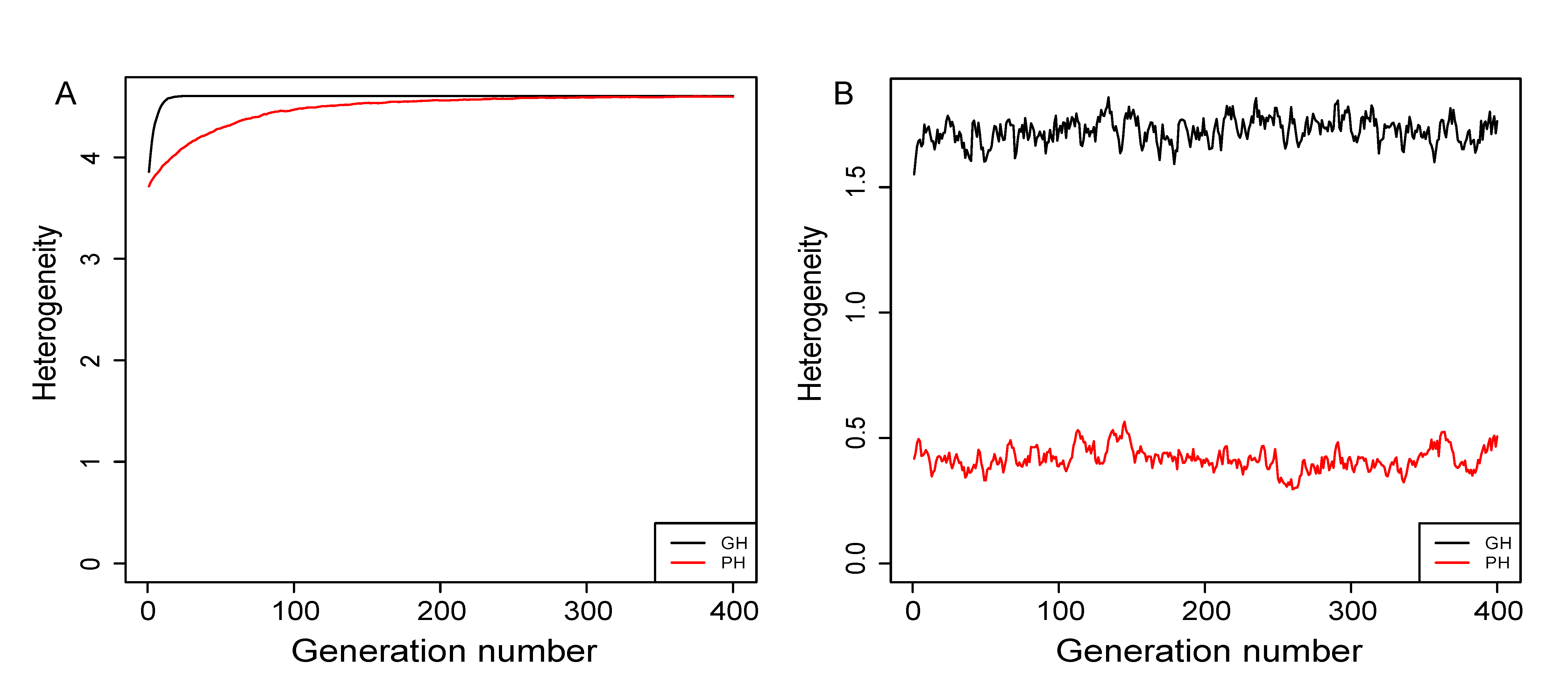

Supplement: S1 Fig — Evolutionary process of genotypic (GH) and phenotypic (PH) heterogeneities in an in silico simulation. The population size in the simulation is 100, mutation rate is 0.1, mutational robustness is 0.1 and noise level is 0.1. A). When no selective constraint was involved, both GH and PH could reach to the upper limit of heterogeneities for the given population, but when the population was subjected to stabilizing selection B), the maximal heterogeneities for both genotype and phenotype were both substantially reduced, and PH was much less than GH. (TIF) [file pone.0116167.s001.tif]

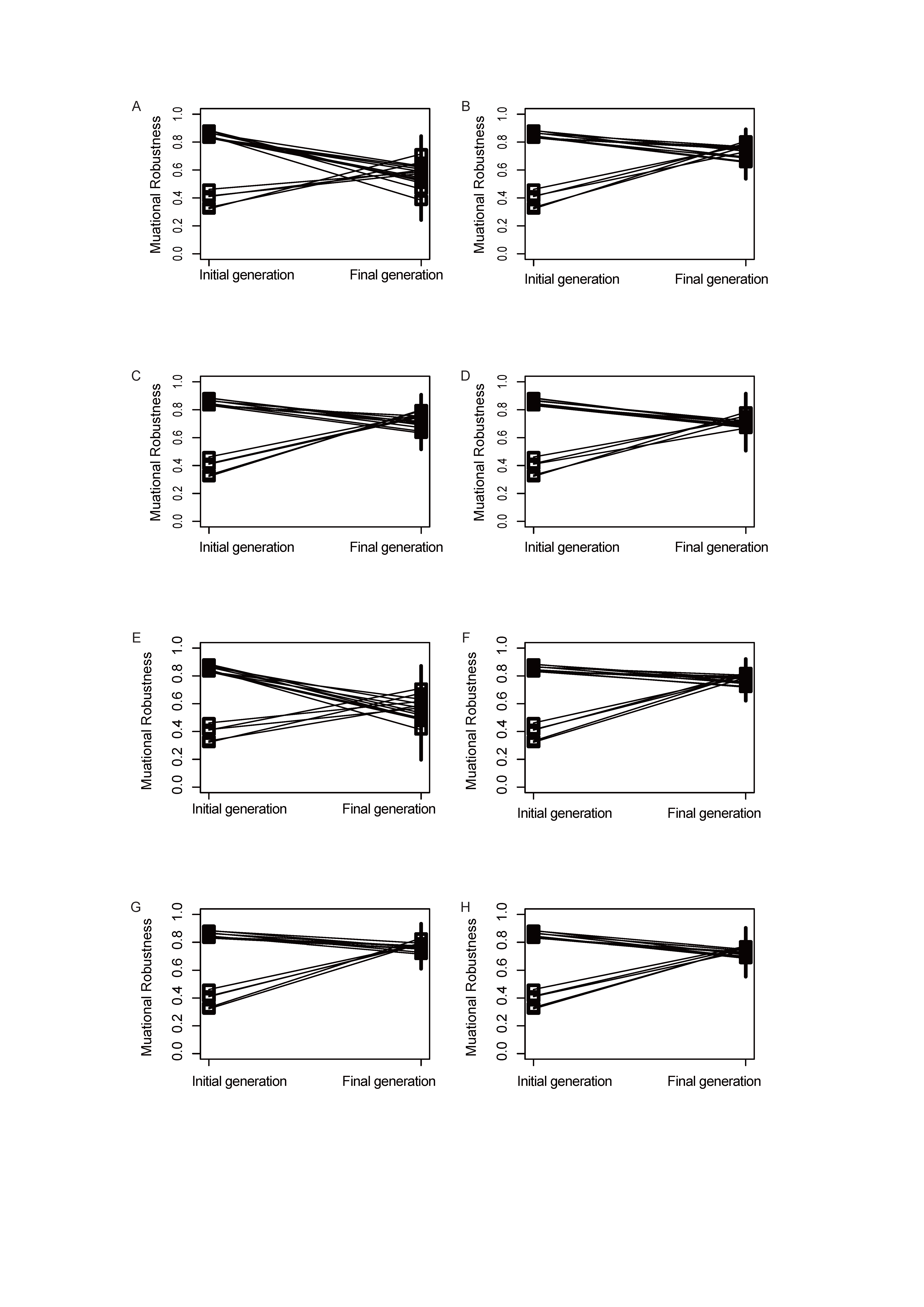

Supplement: S2 Fig — Convergent evolution of mutational robustness of GRNs. The dot pairs linked by solid lines represent the actual mutational robustness at the beginning and end of simulations for a group of GRNs with identical population sizes. The population size is 50, and mutation rates are A) 0.001, B) 0.05, C) 0.1 and D) 0.2. Population size is 200, and mutation rates are E) 0.001, F) 0.05, G) 0.1 and H) 0.2. (TIF) [file pone.0116167.s002.tif]

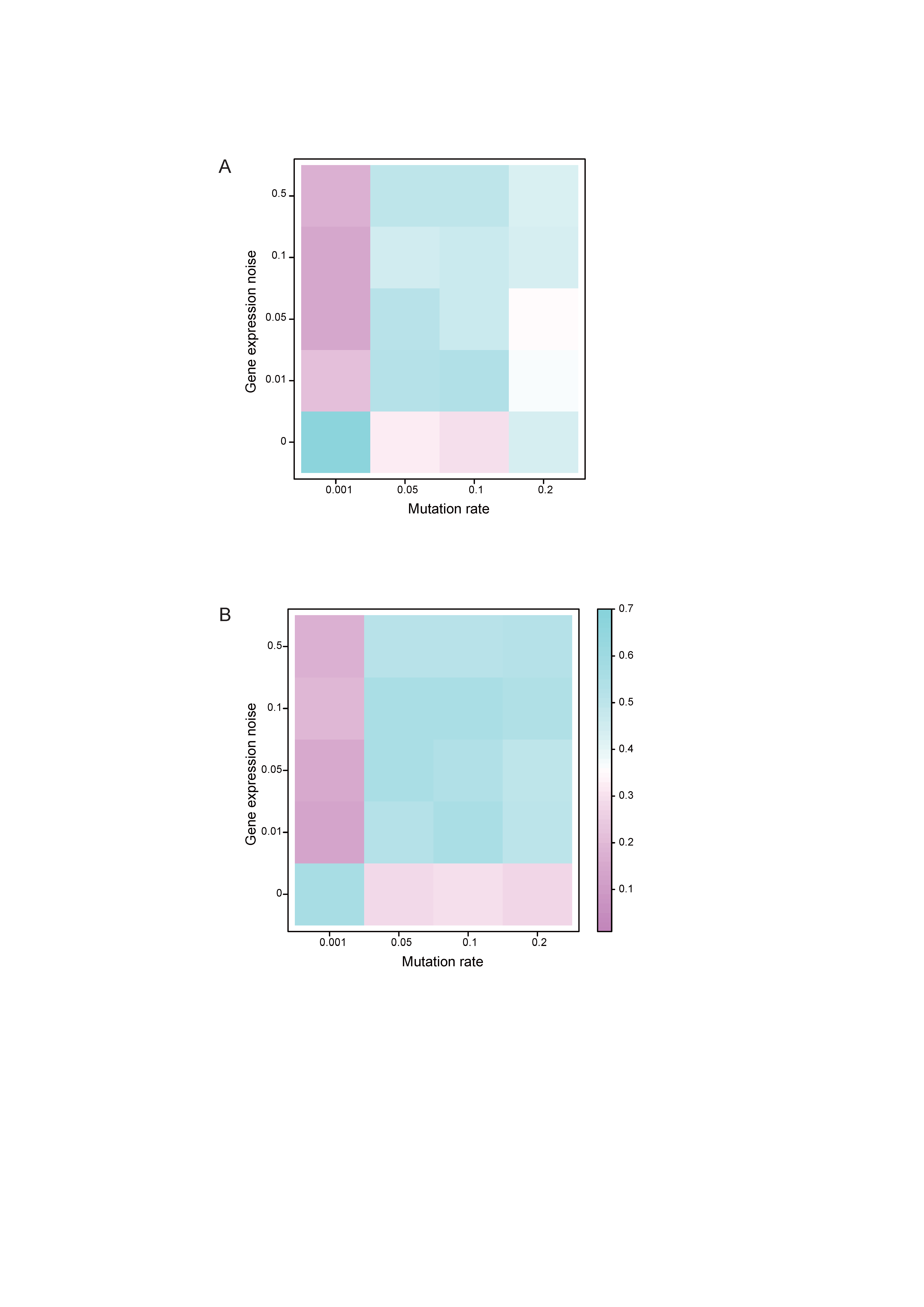

Supplement: S3 Fig — The interaction among mutation, mutational robustness and gene expression noise. Each square represents a set of simulations with identical gene expression noise level and mutation rate. The color in each square index was defined as the proportion of simulation pairs indicating that a population with higher mutational robustness would be more phenotypically heterogeneous than a population with lower mutational robustness after stabilizing selection. The population size is A) 50 and B) 200. (TIF) [file pone.0116167.s003.tif]

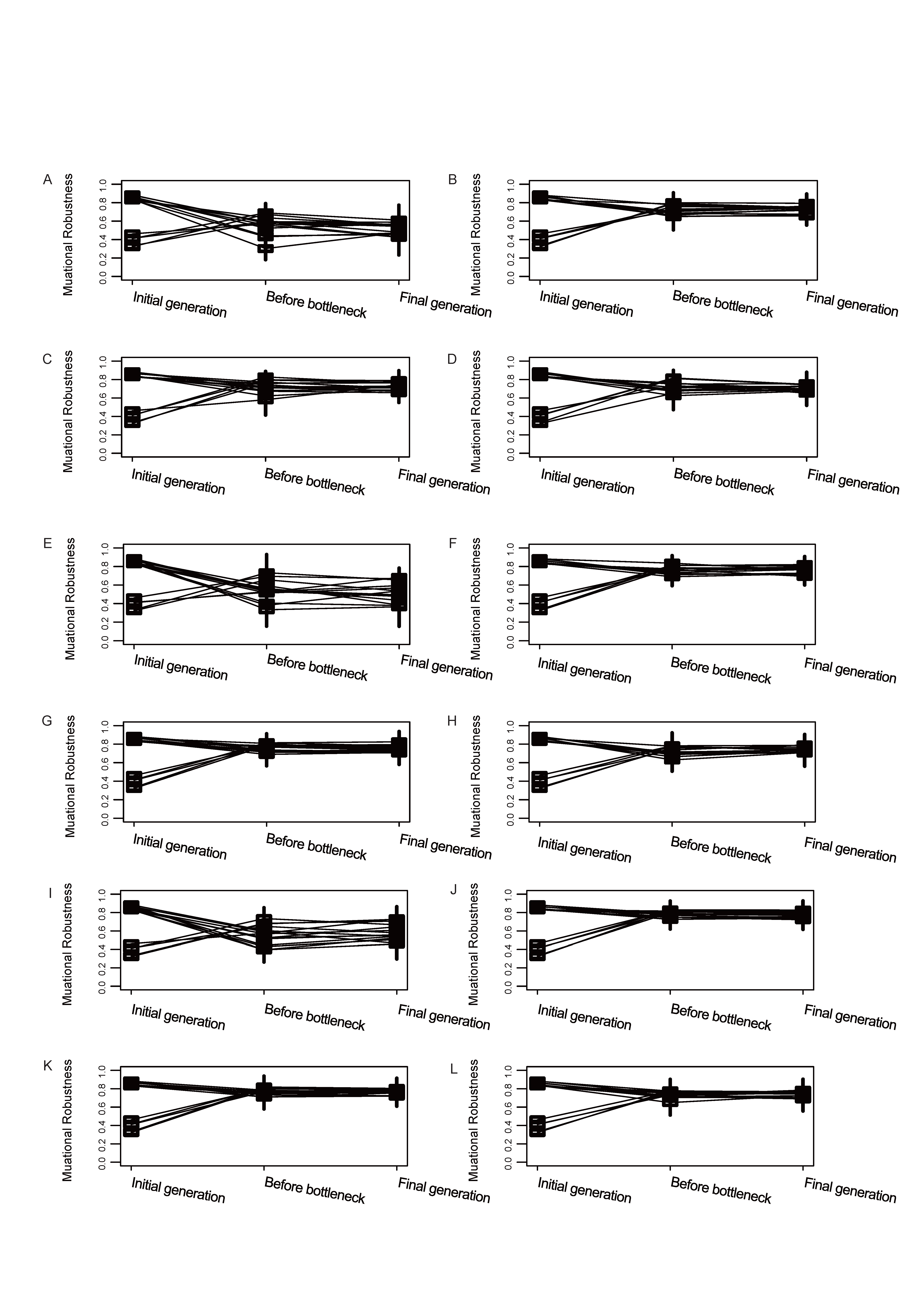

Supplement: S4 Fig — The effect of type I population bottleneck on the evolution of mutational robustness of GRNs. The dots linked by solid lines represent the actual mutational robustness at the beginning generation, before introduction of the bottleneck and after finishing simulations for a group of GRNs with identical population sizes. Population size is 50, and mutation rates are A) 0.001, B) 0.05, C) 0.1 and D) 0.2. Population size is 100, and mutation rates are E) 0.001, F) 0.05, G) 0.1 and H) 0.2. Population size is 200, and mutation rates are I) 0.001, J) 0.05, K) 0.1 and L) 0.2. (TIF) [file pone.0116167.s004.tif]

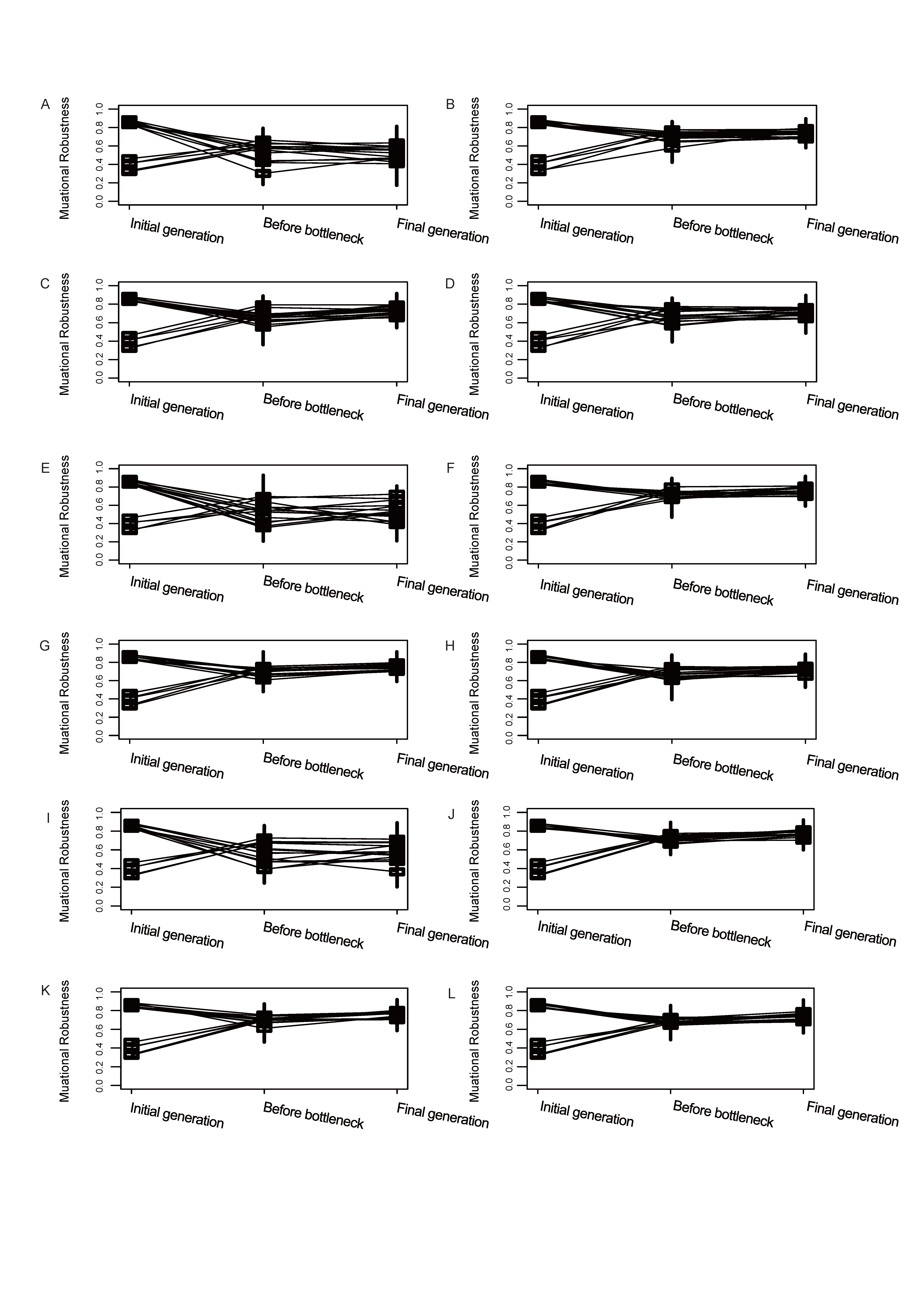

Supplement: S5 Fig — The effect of type II population bottleneck on the evolution of mutational robustness of GRNs. The dots linked by solid lines represent the actual mutational robustness at the beginning generation, before introduction of the bottleneck and after finishing simulations for a group of GRNs with identical population sizes. The population size is 50, and mutation rates are A) 0.001, B) 0.05, C) 0.1 and D) 0.2. Population size is 100, and mutation rates are E) 0.001, F) 0.05, G) 0.1 and H) 0.2. Population size is 200, and mutation rates are I) 0.001, J) 0.05, K) 0.1 and L) 0.2. (TIF) [file pone.0116167.s005.tif]

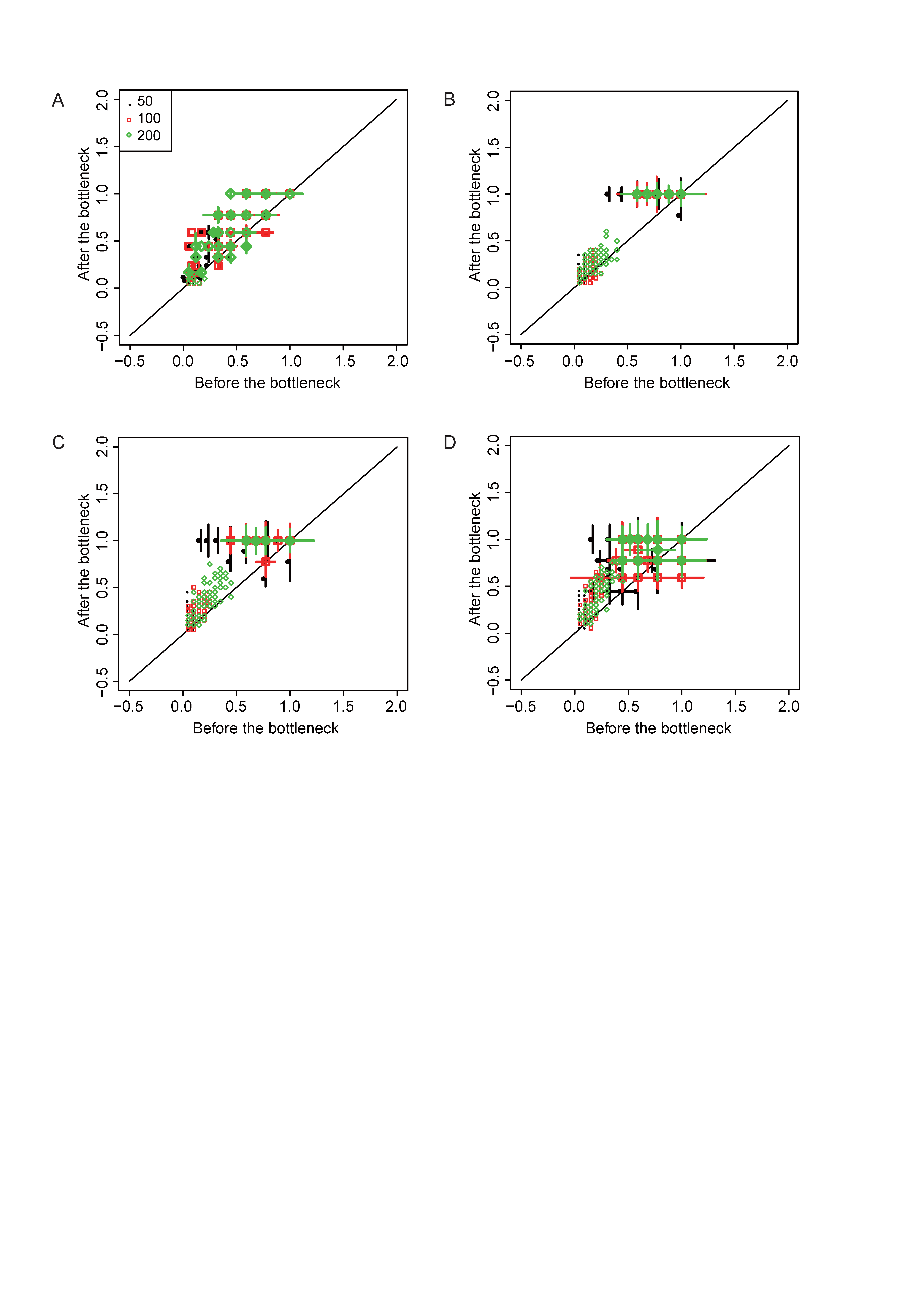

Supplement: S6 Fig — Fitness gain and number of potential oncogene changes before and after passing through type II population bottleneck. Each boxplots represents median and standard deviation of maximal fitness gain before and after the bottleneck for 50 simulation repeats. The dots represent the percentage of potential oncogenes before and after the bottleneck (see main text for definition). Gene expression noise level is 0, population size is indicated in color, and mutation rates are A) 0.001, B) 0.05, C) 0.1 and D) 0.2. (TIF) [file pone.0116167.s006.tif]

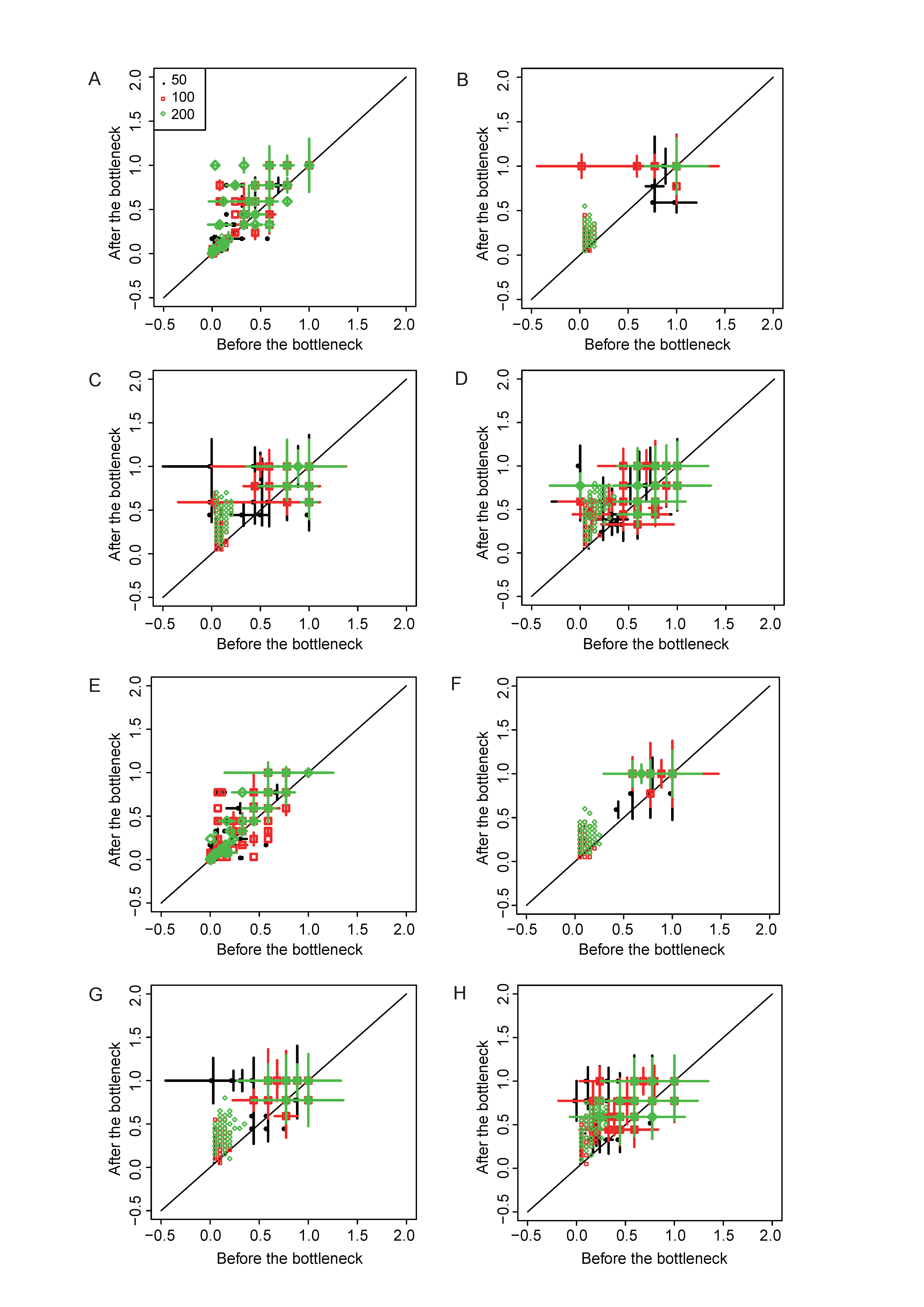

Supplement: S7 Fig — Fitness gain and number of potential oncogene changes before and after passing through population bottlenecks. Each boxplot represents median and standard deviation of maximal fitness gain before and after the bottleneck for 50 simulation repeats. The dots represent the percentage of potential oncogenes before and after the bottleneck (see main text for definition). Gene expression noise level is 0.01, and population size is indicated in color. For type I bottlenecks, the mutation rates are A) 0.001, B) 0.05, C) 0.1 and D) 0.2. For type II bottlenecks, the mutation rates are E) 0.001, F) 0.05, G) 0.1 and H) 0.2. (TIF) [file pone.0116167.s007.tif]

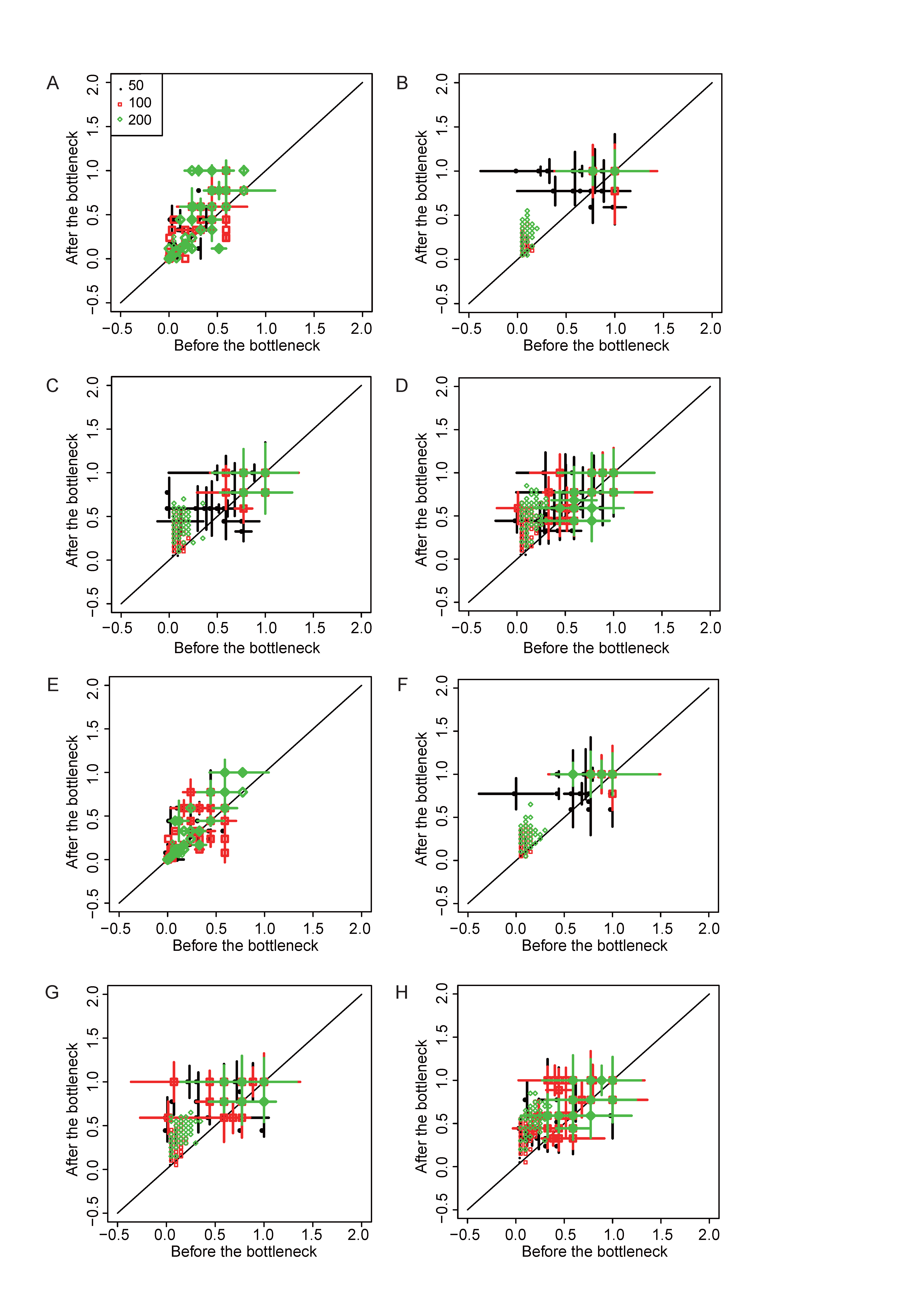

Supplement: S8 Fig — Fitness gain and number of potential oncogene changes before and after passing through population bottlenecks. Each boxplot represents median and standard deviation of maximal fitness gain before and after the bottleneck for 50 simulation repeats. The dots represent the percentage of potential oncogenes before and after the bottleneck (see main text for definition). Gene expression noise level is 0.5, and population size is indicated in color. For type I bottlenecks, the mutation rates are A) 0.001, B) 0.05, C) 0.1 and D) 0.2. For type II bottlenecks, the mutation rates are E) 0.001, F) 0.05, G) 0.1 and H) 0.2. (TIF) [file pone.0116167.s008.tif]
