# Supplementary material for: The Evolution of Heterogeneities Altered by Mutational Robustness, Gene Expression Noise and Bottlenecks in Gene Regulatory Networks
Source: PLoS One. 2014 Dec 26;9(12):e116167. doi: 10.1371/journal.pone.0116167 (PMC4277480; doi:10.1371/journal.pone.0116167)
Supplement: S1 Table — The parameters used in the simulations. All the networks were generated with 20 genes, and for each combination of parameters, the simulation was processed for 100,000 generations and repeated 50 times. (DOCX) [file pone.0116167.s009.docx]

| Parameter names | Symbols | Sampled values |
| --- | --- | --- |
| Population size | *S* | 50, 100, 200 |
| Mutation rate | $m$ | 0.001, 0.05, 0.1, 0.2 |
| Noise | $\delta$ | 0, 0.01, 0.05, 0.1, 0.5 |
| Mutational Robustness | $d$ | 0.1, 0.5, 0.8 |

Table S1
