# Supplementary material for: The Evolution of Heterogeneities Altered by Mutational Robustness, Gene Expression Noise and Bottlenecks in Gene Regulatory Networks
Source: PLoS One. 2014 Dec 26;9(12):e116167. doi: 10.1371/journal.pone.0116167 (PMC4277480; doi:10.1371/journal.pone.0116167)
Supplement: S2 Table — PH Difference between pre- and post-bottlenecks in GRN networks. P-values were given by t-test. (DOCX) [file pone.0116167.s010.docx]

| Bn | S | M | $\delta$ | diff | pva |
| --- | --- | --- | --- | --- | --- |
| 1 | 50 | 0.001 | 1 | 0.1205 | 0.0001 |
| 1 | 50 | 0.05 | 1 | 0.0009 | 0.9655 |
| 1 | 50 | 0.1 | 1 | 0.0097 | 0.6929 |
| 1 | 50 | 0.2 | 1 | -0.0124 | 0.4092 |
| 1 | 100 | 0.001 | 1 | 0.0684 | 0.0474 |
| 1 | 100 | 0.05 | 1 | 0.0025 | 0.9104 |
| 1 | 100 | 0.1 | 1 | -0.0174 | 0.4241 |
| 1 | 100 | 0.2 | 1 | -0.0111 | 0.5952 |
| 1 | 200 | 0.001 | 1 | 0.1705 | 0.0000 |
| 1 | 200 | 0.05 | 1 | 0.0552 | 0.0443 |
| 1 | 200 | 0.1 | 1 | 0.0121 | 0.5483 |
| 1 | 200 | 0.2 | 1 | -0.0562 | 0.0288 |
| 1 | 50 | 0.001 | 2 | 0.1258 | 0.0001 |
| 1 | 50 | 0.05 | 2 | -0.0146 | 0.5493 |
| 1 | 50 | 0.1 | 2 | -0.0051 | 0.7881 |
| 1 | 50 | 0.2 | 2 | -0.0110 | 0.4032 |
| 1 | 100 | 0.001 | 2 | 0.1012 | 0.0019 |
| 1 | 100 | 0.05 | 2 | 0.0681 | 0.0010 |
| 1 | 100 | 0.1 | 2 | 0.0429 | 0.0842 |
| 1 | 100 | 0.2 | 2 | 0.0142 | 0.4341 |
| 1 | 200 | 0.001 | 2 | 0.0994 | 0.0030 |
| 1 | 200 | 0.05 | 2 | 0.0330 | 0.1066 |
| 1 | 200 | 0.1 | 2 | -0.0170 | 0.3900 |
| 1 | 200 | 0.2 | 2 | 0.0141 | 0.5604 |
| 1 | 50 | 0.001 | 3 | 0.0670 | 0.0354 |
| 1 | 50 | 0.05 | 3 | 0.0238 | 0.2489 |
| 1 | 50 | 0.1 | 3 | 0.0189 | 0.3022 |
| 1 | 50 | 0.2 | 3 | -0.0087 | 0.5089 |
| 1 | 100 | 0.001 | 3 | 0.1463 | 0.0000 |
| 1 | 100 | 0.05 | 3 | -0.0074 | 0.7874 |
| 1 | 100 | 0.1 | 3 | 0.0209 | 0.3205 |
| 1 | 100 | 0.2 | 3 | 0.0145 | 0.4710 |
| 1 | 200 | 0.001 | 3 | 0.0724 | 0.0585 |
| 1 | 200 | 0.05 | 3 | 0.0434 | 0.0203 |
| 1 | 200 | 0.1 | 3 | -0.0330 | 0.1717 |
| 1 | 200 | 0.2 | 3 | 0.0308 | 0.0604 |
| 1 | 50 | 0.001 | 4 | 0.0503 | 0.1193 |
| 1 | 50 | 0.05 | 4 | -0.0280 | 0.2488 |
| 1 | 50 | 0.1 | 4 | -0.0068 | 0.7642 |
| 1 | 50 | 0.2 | 4 | 0.0006 | 0.9703 |
| 1 | 100 | 0.001 | 4 | 0.1570 | 0.0000 |
| 1 | 100 | 0.05 | 4 | 0.0074 | 0.7577 |
| 1 | 100 | 0.1 | 4 | -0.0018 | 0.9270 |
| 1 | 100 | 0.2 | 4 | -0.0199 | 0.2746 |
| 1 | 200 | 0.001 | 4 | 0.0936 | 0.0194 |
| 1 | 200 | 0.05 | 4 | 0.0715 | 0.0011 |
| 1 | 200 | 0.1 | 4 | -0.0213 | 0.2313 |
| 1 | 200 | 0.2 | 4 | -0.0254 | 0.2348 |
| 1 | 50 | 0.001 | 5 | 0.1017 | 0.0010 |
| 1 | 50 | 0.05 | 5 | -0.0639 | 0.0174 |
| 1 | 50 | 0.1 | 5 | 0.0150 | 0.4117 |
| 1 | 50 | 0.2 | 5 | 0.0109 | 0.3543 |
| 1 | 100 | 0.001 | 5 | 0.1343 | 0.0003 |
| 1 | 100 | 0.05 | 5 | -0.0236 | 0.3602 |
| 1 | 100 | 0.1 | 5 | -0.0141 | 0.5941 |
| 1 | 100 | 0.2 | 5 | -0.0201 | 0.2821 |
| 1 | 200 | 0.001 | 5 | 0.1065 | 0.0050 |
| 1 | 200 | 0.05 | 5 | 0.0169 | 0.4780 |
| 1 | 200 | 0.1 | 5 | 0.0067 | 0.7249 |
| 1 | 200 | 0.2 | 5 | -0.0037 | 0.8494 |
| 2 | 50 | 0.001 | 1 | 0.1205 | 0.0001 |
| 2 | 50 | 0.05 | 1 | 0.0009 | 0.9655 |
| 2 | 50 | 0.1 | 1 | 0.0097 | 0.6929 |
| 2 | 50 | 0.2 | 1 | -0.0124 | 0.4092 |
| 2 | 100 | 0.001 | 1 | 0.0684 | 0.0474 |
| 2 | 100 | 0.05 | 1 | 0.0025 | 0.9104 |
| 2 | 100 | 0.1 | 1 | -0.0174 | 0.4241 |
| 2 | 100 | 0.2 | 1 | -0.0111 | 0.5952 |
| 2 | 200 | 0.001 | 1 | 0.1705 | 0.0000 |
| 2 | 200 | 0.05 | 1 | 0.0552 | 0.0443 |
| 2 | 200 | 0.1 | 1 | 0.0121 | 0.5483 |
| 2 | 200 | 0.2 | 1 | -0.0562 | 0.0288 |
| 2 | 50 | 0.001 | 2 | 0.1258 | 0.0001 |
| 2 | 50 | 0.05 | 2 | -0.0146 | 0.5493 |
| 2 | 50 | 0.1 | 2 | -0.0051 | 0.7881 |
| 2 | 50 | 0.2 | 2 | -0.0110 | 0.4032 |
| 2 | 100 | 0.001 | 2 | 0.1012 | 0.0019 |
| 2 | 100 | 0.05 | 2 | 0.0681 | 0.0010 |
| 2 | 100 | 0.1 | 2 | 0.0429 | 0.0842 |
| 2 | 100 | 0.2 | 2 | 0.0142 | 0.4341 |
| 2 | 200 | 0.001 | 2 | 0.0994 | 0.0030 |
| 2 | 200 | 0.05 | 2 | 0.0330 | 0.1066 |
| 2 | 200 | 0.1 | 2 | -0.0170 | 0.3900 |
| 2 | 200 | 0.2 | 2 | 0.0141 | 0.5604 |
| 2 | 50 | 0.001 | 3 | 0.0670 | 0.0354 |
| 2 | 50 | 0.05 | 3 | 0.0238 | 0.2489 |
| 2 | 50 | 0.1 | 3 | 0.0189 | 0.3022 |
| 2 | 50 | 0.2 | 3 | -0.0087 | 0.5089 |
| 2 | 100 | 0.001 | 3 | 0.1463 | 0.0000 |
| 2 | 100 | 0.05 | 3 | -0.0074 | 0.7874 |
| 2 | 100 | 0.1 | 3 | 0.0209 | 0.3205 |
| 2 | 100 | 0.2 | 3 | 0.0145 | 0.4710 |
| 2 | 200 | 0.001 | 3 | 0.0724 | 0.0585 |
| 2 | 200 | 0.05 | 3 | 0.0434 | 0.0203 |
| 2 | 200 | 0.1 | 3 | -0.0330 | 0.1717 |
| 2 | 200 | 0.2 | 3 | 0.0308 | 0.0604 |
| 2 | 50 | 0.001 | 4 | 0.0503 | 0.1193 |
| 2 | 50 | 0.05 | 4 | -0.0280 | 0.2488 |
| 2 | 50 | 0.1 | 4 | -0.0068 | 0.7642 |
| 2 | 50 | 0.2 | 4 | 0.0006 | 0.9703 |
| 2 | 100 | 0.001 | 4 | 0.1570 | 0.0000 |
| 2 | 100 | 0.05 | 4 | 0.0074 | 0.7577 |
| 2 | 100 | 0.1 | 4 | -0.0018 | 0.9270 |
| 2 | 100 | 0.2 | 4 | -0.0199 | 0.2746 |
| 2 | 200 | 0.001 | 4 | 0.0936 | 0.0194 |
| 2 | 200 | 0.05 | 4 | 0.0715 | 0.0011 |
| 2 | 200 | 0.1 | 4 | -0.0213 | 0.2313 |
| 2 | 200 | 0.2 | 4 | -0.0254 | 0.2348 |
| 2 | 50 | 0.001 | 5 | 0.1017 | 0.0010 |
| 2 | 50 | 0.05 | 5 | -0.0639 | 0.0174 |
| 2 | 50 | 0.1 | 5 | 0.0150 | 0.4117 |
| 2 | 50 | 0.2 | 5 | 0.0109 | 0.3543 |
| 2 | 100 | 0.001 | 5 | 0.1343 | 0.0003 |
| 2 | 100 | 0.05 | 5 | -0.0236 | 0.3602 |
| 2 | 100 | 0.1 | 5 | -0.0141 | 0.5941 |
| 2 | 100 | 0.2 | 5 | -0.0201 | 0.2821 |
| 2 | 200 | 0.001 | 5 | 0.1065 | 0.0050 |
| 2 | 200 | 0.05 | 5 | 0.0169 | 0.4780 |
| 2 | 200 | 0.1 | 5 | 0.0067 | 0.7249 |
| 2 | 200 | 0.2 | 5 | -0.0037 | 0.8494 |

Table S2
